# Orthodontic Treatment and Healthcare Goals: Evaluation of Multibrackets Treatment Results Using PAR Index (Peer Assessment Rating)

**DOI:** 10.3390/healthcare8040473

**Published:** 2020-11-10

**Authors:** Maria Francesca Sfondrini, Paolo Zampetti, Giulia Luscher, Paola Gandini, José Luís Gandía-Franco, Andrea Scribante

**Affiliations:** 1 Unit of Orthodontics and Paediatric Dentistry, Section of Dentistry-Department of Clinical, Surgical, Diagnostic and Paediatric Sciences, University of Pavia, Pavia, Piazzale Golgi 2, 27100 Pavia, Italy; francesca.sfondrini@unipv.it (M.F.S.); giulia.luscher01@universitadipavia.it (G.L.); paola.gandini@unipv.it (P.G.); 2 Section of Dentistry-Department of Clinical, Surgical, Diagnostic and Paediatric Sciences, University of Pavia, Pavia, Piazzale Golgi 2, 27100 Pavia, Italy; paolo.zampetti@unipv.it; 3 Tenured Lecturer, Stomatology Department, Faculty of Medicine and Dentistry, University of Valencia, 46010 Valencia, Spain; chelugf@gmail.com

**Keywords:** dentistry, orthodontics, therapy, peer assessment rating, PAR, treatment, outcome, quality, healthcare, bracket

## Abstract

Background: The evaluation of orthodontic treatment outcomes using an objective method is important in order to maintain high treatment quality and final healthcare of patients. It allows professionals and university students to raise the level of the therapy. The aim of this study was to assess the orthodontic treatment outcomes in an Italian postgraduate School of Orthodontics using Peer Assessment Rating (PAR) Index. Methods: A sample of 50 patients treated in a postgraduate program was randomly selected. PAR index was used to assess pre-treatment and post-treatment study casts by two different examiners. The influence of different variables such as gender, treatment method, and need for extraction was statistically analyzed. Results: The average numerical reduction of PAR between the beginning and the end of the treatment was 18.74 (CI 95% 16.53–20.95), while the percentage reduction was 94.8% (CI 95% 91.91–97.68). All cases improved: 8% of patients resulted in the improved category, while 92% of them were in the greatly improved group. Conclusions: According to PAR index, the results showed that patients received a high-standard therapy. None of the factors studied influenced significantly the treatment outcomes.

## 1. Introduction

The evaluation of orthodontic treatment outcomes has traditionally been accomplished using the experience and subjective opinions of clinicians [1]. However, since the nineties, several indices have been specifically developed to objectively evaluate the healthcare results by analyzing the quality of the treatment [2]. These indices compare pre- and post-treatment data to determine the outcome of orthodontic therapy [3] and to improve the quality of future treatments [4]. The most commonly used index to assess orthodontic success is Peer Assessment Rating Index (PAR), which was developed to measure how much a patient deviates from normal occlusion and alignment [2]. This index has been used to evaluate the effects of therapy in different circumstances: the use of fixed and removable devices [5], the comparison of orthodontic treatment between private practices and orthodontics schools [6], the evaluation of the occlusal stability after orthodontic treatment [7], early treatments [8], and the outcome of orthognathic surgery [9].

**Figure 1:**
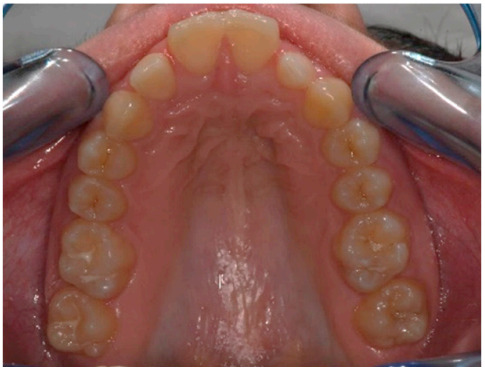
Upper anterior segments.

**Figure 2:**
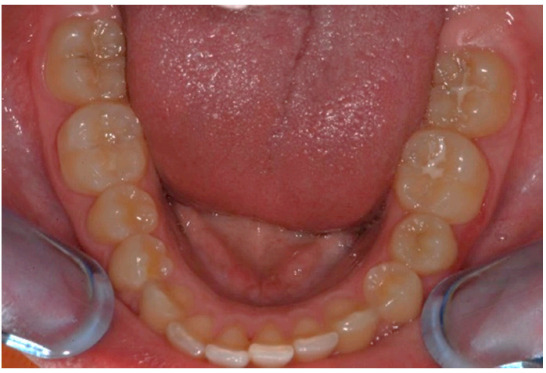
Lower anterior segments.

**Figure 3:**
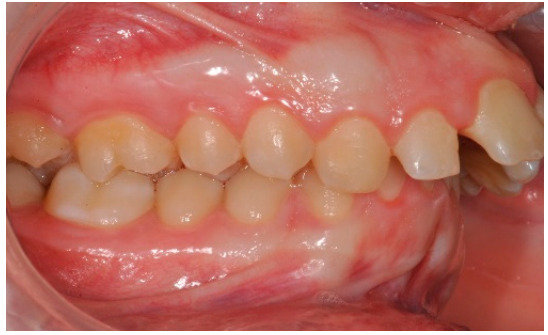
Right buccal segments relationship.

**Figure 4:**
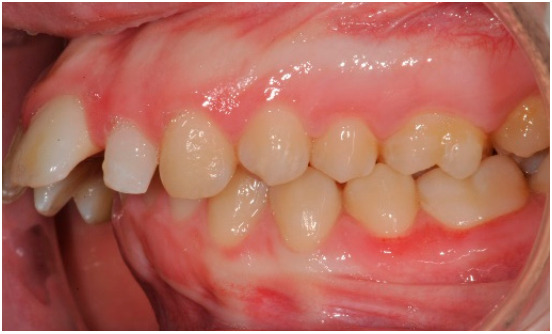
Left buccal segments relationship.

**Figure 5:**
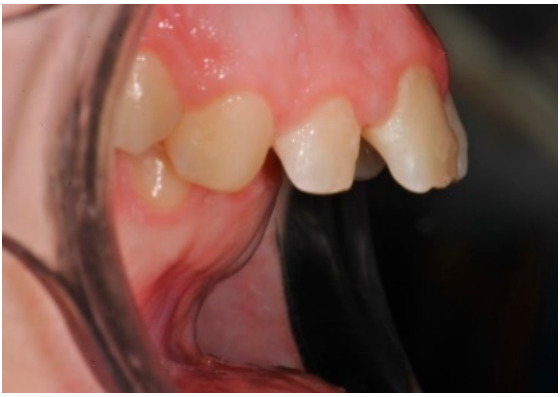
Overjet.

**Figure 6:**
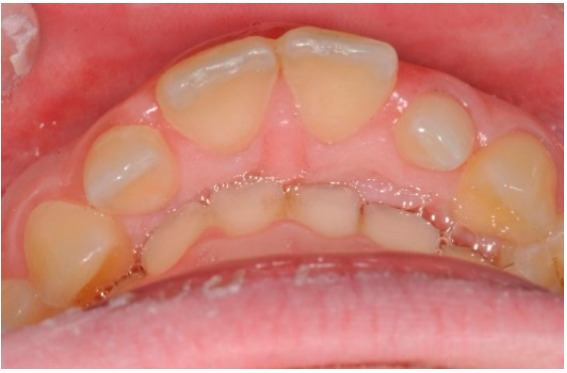
Overbite.

**Figure 7:**
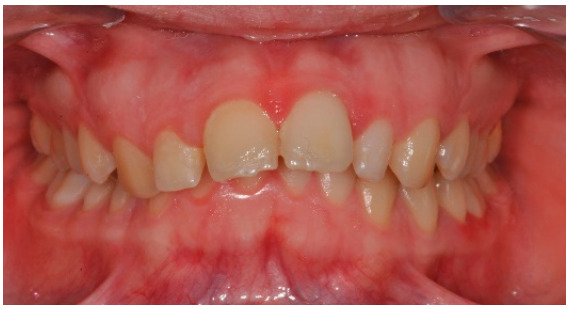
Midline.

As shown in Figure 1, Figure 2, Figure 3, Figure 4, Figure 5, Figure 6 and Figure 7, PAR index consists of seven components: upper and lower anterior segments, overbite, overjet, midline, and right and left buccal segments relationship [10]. These variables, acquired from pre- and post-treatment study casts, are added to each other to generate a total pre-therapy and post-therapy score. The difference between initial and final PAR reflects the degree of improvement and therefore the success of the treatment. The more the score tends to zero (which correspond to an ideal occlusion), the less the deviation from a normal condition [2].

This index has been shown to have good intra- and inter-examiner reliability; moreover, it allows the assessment of orthodontics outcomes in a standardized way because it divides the patients into three categories depending on the improvement induced by the treatment: worsening/no difference (reduction in PAR score less than 30%); improvement (reduction greater than or equal to 30%); great improvement (reduction more than 22 points or greater than 70%) [2].

In accordance with the general classification criteria of the index, to demonstrate high-standard treatments in terms of healthcare gain for the patient, the sample should be composed of a percentage greater than 70% of the cases that shows improvement, more than 40% of them should fall into the category of great improvement, and only a negligible part of cases (up to a maximum of 5%) should belong to the worsening/no difference group. The greater the mean reduction in PAR score, the higher the standard of orthodontics achieved [2].

In this perspective, the objectives of this study were as follows:

(1) To evaluate the quality of the orthodontic treatment performed by students of a postgraduate orthodontic program, using the weighted PAR.
(2) To determine whether the outcome of treatment (in terms of change in PAR) is related to the following factors: gender, treatment technique, dental extractions.

## 2. Material and Methods

The sample for this research was obtained from patients treated with upper and lower multibrackets appliances during the last ten years at the postgraduate School of Orthodontics at University of Pavia (Italy). Unit Internal Review Board approved the study (2019-0925). Fifty patients with different types of malocclusion were randomly selected from the case file by the examiner using the software Excel (v. 15, Microsoft, Redmond, Washington, DC, USA). Sample size calculation was performed with a computer software (Sample Size Calculator, ClinCalc LLC, Indianapolis, IN, USA) based on previous studies [1,10] with Alpha = 0.05, Power = 90%, and a continuous primary endpoint. It required 50 total participants.

**Figure 8:**
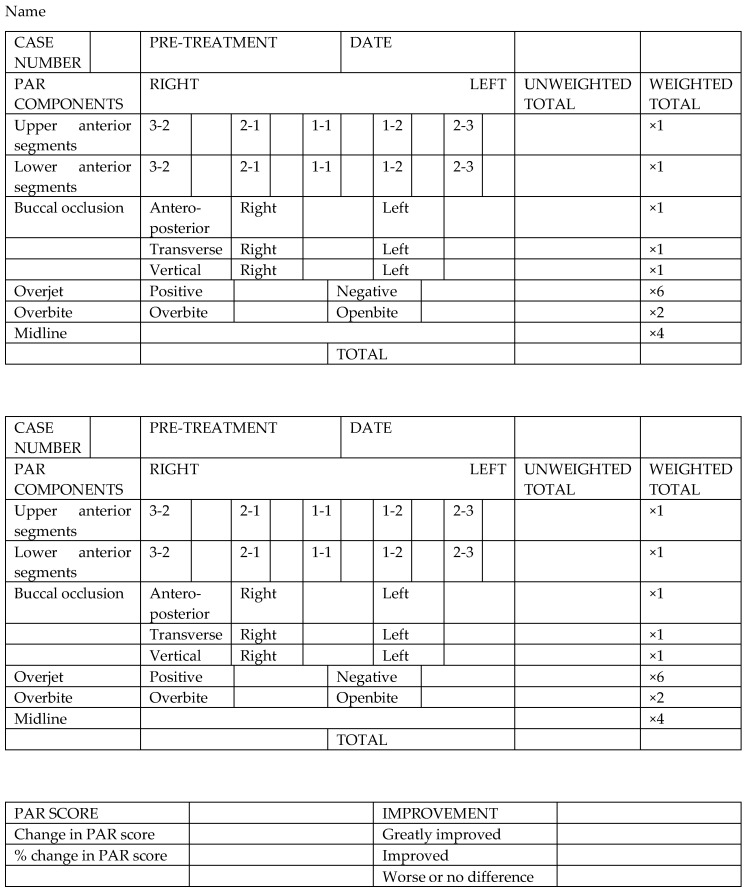
Form used to perform Peer Assessment Rating (PAR) score.

The main information collected was gender, date of birth, therapy start and end date, age at the beginning of treatment, duration of treatment, treatment technique (straight wire or self-ligating) need for extractions, and level of cooperation. Patients with incomplete diagnostical records (pre- and post-treatment casts, medical chart) and patients treated with orthognathic surgery or with one-arch appliance were excluded. The measurements were performed, using an orthodontic digital caliper (G&H Ortodontics, Franklin, TN, USA), on hard plaster type III pre-treatment casts (at the case study or before the acceptance of the treatment) and on post-treatment casts (at debonding), articulated together. PAR score was generated by analyzing the following variables: alignment of the upper anterior segment, alignment of the lower anterior segment, right and left occlusion (including transverse, vertical, and sagittal alterations), overjet, overbite, and midline. In order to obtain the total score, the weighted PAR, which attributes a value to a single variable, was used. The weighted values for each variable were added to generate a total pre-therapy and a total post-treatment score; by comparing these two data, the numerical and percentage reduction of PAR was obtained (the form used to perform the PAR score is shown in Figure 8). Based on the results achieved, patients were divided into three categories depending on the degree of improvement induced by orthodontic treatment (no difference or worsening/improvement/great improvement). The error associated with PAR recording method was evaluated from double measurements, one month apart from each other, by the same examiner. The Intraclass Correlation Coefficient (ICC) was used to analyze intra-examiner reliability in order to evaluate the validity of the method.

All cases were evaluated by two examiners. Inter-examiner reliability was also assessed with the Intraclass Correlation Coefficient (ICC). In order to estimate the relationship between the variables and PAR numerical and percentage reduction, nonparametric tests were performed; Mann–Whitney U test was applied to the categorical variables (gender, type of technique, and extraction) at a significance level of *p* < 0.05.

Statistical analysis was carried out using the software R (R version 3.1.3, R Development Core Team, R Foundation for Statistical Computing, Vienna, Austria).

## 3. Results

The sample of 50 patients consisted of 22 females (44%) and 28 males (56%). The range of age was between 14 years and 10 months and 42 years. The average pre-treatment age was 14 years and 1 month (169 months, CI 95% 155–183), while the average treatment duration was 2 years and 7 months (31 months, CI 95% 28–33). The techniques used were straight wire system in 43 cases (86% of patients) and self-ligating appliance in 7 cases (14% of patients). The extraction solution was performed in 5 cases (10% of the patients).

**Figure 9:**
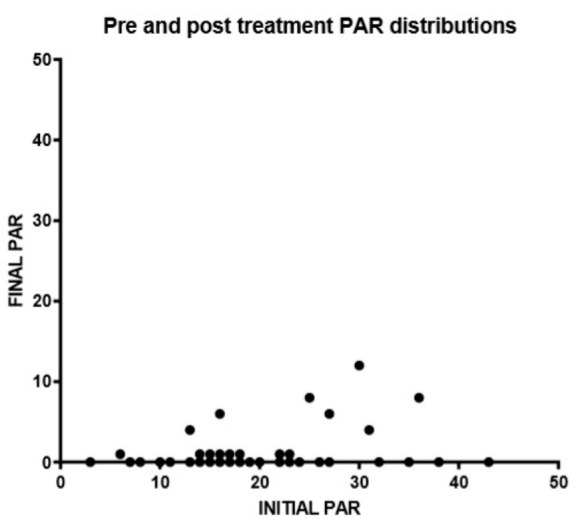
Relation between pre-treatment and post-treatment PAR scores.

**Figure 10:**
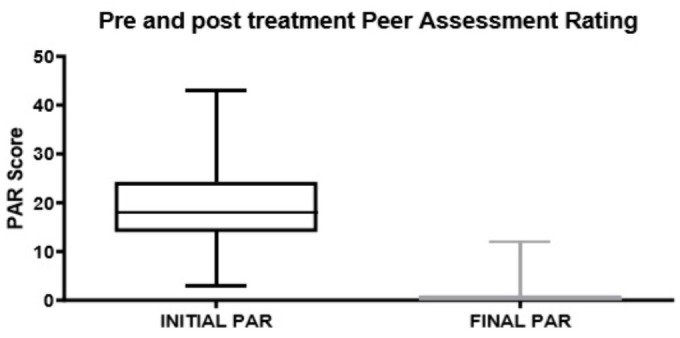
Histogram of pre-treatment and post-treatment PAR scores.

A summary of the results is reported in Table 1. As shown in Figure 9 and Figure 10, the mean pre-treatment PAR score was 19.88 (CI 95% 17.58–22.18) and the mean post-treatment PAR score was 1.14 (CI 95% 0.43–1.85). The difference between pre-treatment and post-treatment scores was significant (*p* < 0.05). The average change value of PAR between the beginning and end of orthodontic treatment was 18.74 (CI 95% 16.53–20.95), while the average percentage reduction was 94.80% (CI 95% 91.91–97.68). No correlation between duration of therapy and PAR index modification was reported in the present study (*p* > 0.05).

None of the categorical variables studied (gender, type of technique, extraction, treatment duration) influenced significantly the results of the treatment (*p* > 0.05).

Patients were divided into three categories based on the reduction in PAR score:

1. Worsening or no difference: 0% of patients.
2. Improvement: 8% of patients.
3. Great improvement: 92% of patients.

Both the Intraclass Correlation Coefficient and the Inter-examiner Correlation Coefficient showed excellent reliability, respectively 0.92 and 0.94.

## 4. Discussion

The sample analyzed in this study was quite similar in the percentage distribution of males and females to other studies [11,12,13]. Moreover, the results of numerical and percentage change of PAR did not alter with patient sex, as found by other researchers [10].

The range of age was between 14 years and 10 months and 42 years; the average pre-treatment age was approximately 14 years and one month, similar to that of Dyken, Onyeaso, and Yang-Powers [1,3,14] and lower than that obtained by a previous report [10]; however, in this last study, despite this higher average value, the 60% of patients underwent treatment between 10 and 15 years old. Furthermore, no association between initial age and treatment outcome was found.

The average treatment duration was 31 months, according to the results obtained from other similar studies [10,15,16]. The relationship between duration of therapy and PAR index modifications was not found to be relevant. Some authors have argued that longer treatment increases the likelihood of producing worse results as the patient’s collaboration decreases [17,18,19]. Pinskaya, for example, stated that long-term treatment (up to thirty-nine months) is accompanied by lower clinical results and a progressive deterioration in the quality of therapy due to the “patient burn out”, with a gradual reduction of patient cooperation [15]. Although other researchers have reported conflicting results [20], it should be stressed that long-term treatment increases the risk of iatrogenic injury. In this perspective, it becomes important to distinguish between cases where therapy extends due to lack of collaboration by the patient and those in which the orthodontist decides to enhance the active treatment phase in order to obtain better results [10].

Regarding the relationship between results and different treatment techniques (MBT-McLaughlin/Bennett/Trevisi and self-liganting), no statistical significance was found, in accordance with previous studies [21,22,23] and with the research of a previous study, which considered five different techniques (Standard, Bidimensional, MBT, Tip-edge, and Smart-clip) [10]. Therefore, different types of treatment can produce similar clinical results.

Another variable taken into account is the need for dental extraction: the percentage of extraction cases in this sample (10%) was lower than in the samples analyzed in other studies [10,13,15] due to the interceptive treatment that most patients underwent. In all these studies, no statistically significant relationship was found between extraction and treatment outcome; therefore, the therapy can be satisfactorily completed independently of the extractive or nonextractive choice [24].

The need for an adequate level of collaboration by the patient and for a good oral hygiene is fundamental and therefore it was required for all patients, unlike the study of Gandia in which it was considered necessary for 80% of the subjects examined [10]. This aspect is crucial to allow the prosecution and conclusion of orthodontic treatment, which would otherwise be suspended prematurely for lack of cooperation. In fact, several authors have agreed to affirm that the absence of a correct oral hygiene is the major cause of failure or poor success of therapy [20]. In particular, Beckwith et al. have conducted a study about the factors that may affect the duration of orthodontic treatment, concluding that about half of the variations can be explained by patient collaboration [25]. In addition, problematic patients (such as those who are not collaborative in meeting appointments, those who do not cooperate with elastics, those who detach many brackets, or those who have an insufficient degree of oral hygiene) do not show any improvement in the final results if they undergo an additional treatment period [15,26,27,28,29]. This fact, therefore, emphasizes that often the interruption of orthodontic therapy is a clinical decision justified by the safeguarding of the patient’s interest.

The mean pre-treatment PAR value was 19.88, similar to that found by other authors [5,16] and slightly lower than that obtained in the studies of Dyken [1] and Gonzales-Gil-De-Bernabé [10]. 

The mean post-treatment PAR value was 1.14, lower than that obtained in other studies [5,10,30]. This value is due to the fact that in a high percentage of cases it is possible to achieve a perfect alignment of the frontal sector, a coincidence of the upper and lower midlines, and physiological values of overjet and overbite. However, in agreement with the results obtained from the study of Al Yami et al., it seems more difficult to obtain a perfect lateral occlusion, since this parameter is closely dependent on the patient’s collaboration in carrying elastics [31].

The average numerical and percentage changes of PAR, obtained from the initial and final mean values, were respectively 18.74% and 94.80%; while the numerical reduction was similar to that obtained in the study of Gandia [10], the percentage reduction appeared to be greater in this research compared with the data in literature: this discrepancy may be attributed to a minimal inter-examiner subjectivity.

Regarding the validity of the method employed, both intra- and inter-examiner reliability showed high levels, respectively 0.92 and 0.94.

The main limitation of the study was represented by the small sample (fifty patients); it should be noted, however, that this randomly selected sample was representative of the cases and that the number of patients was sufficient to achieve the proposed objectives. In future, the subjects examined could be increased in order to make the research even more reliable.

Additionally, evaluating the efficacy of PAR and comparing it to subjective measures of successful orthodontic treatment would be an interesting addition to the present manuscript since to evaluate only the success of the treatment performed in a specific school gives a partial evaluation.

**Figure 11:**
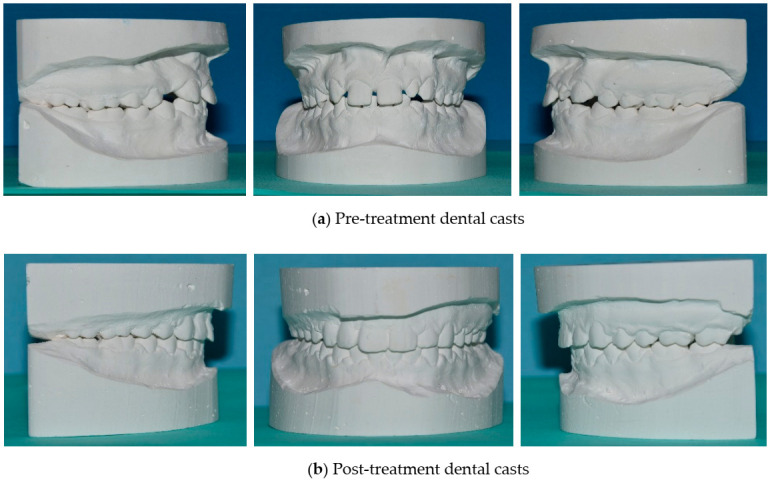
Pre-treatment and post-treatment dental casts.

PAR index allows a general evaluation of the treatment and classifies patients in different categories on the basis of the numerical and percentage reduction between pre-treatment and post-treatment score. The results of this research are objectively significant: if we consider the percentage reduction, all cases improved (100%) and 92% of them underwent a great improvement, while if we evaluate the numerical difference between initial and final PAR, 68% of patients improved as a result of orthodontic therapy and 32% of them greatly improved. Some examples of patient malocclusions showing the difference between the beginning and the end of treatment are represented in Figure 11. The difference in the number of patients falling into the category of great improvement can be attributed to the fact that, based on the numerical reduction, pre-treatment PAR must be more than twenty-two points to allow the patient to be included in this group; however, thirty-four of the fifty patients had initial PAR lower or equal to twenty-two points, as a consequence of interceptive therapy. Therefore, it can be stated that the degree of improvement induced by orthodontic treatment is more reliable if the percentage reduction of the score is analyzed. Compared with other studies [6,16], the results are better because 0% of the patients got worse due to the therapy and because a very high percentage of cases fall under the great improvement category.

The present report evaluated an overview of conventional orthodontic cases. Future research perspective would include studies more focused on the different particular aspects of orthodontic treatment, such as digital orthodontics [32] orthognathic surgery [33], disinclusion [34], or transposition [35] cases. The evaluation of these variables could modify or confirm the present preliminary results. Additionally, the healthcare improvement after the use of emerging technologies such as intraoral scanners [36], lasers [37], miniscrews [38], or experimental CAD/CAM appliances [39] should be evaluated. Future reports and RCTs are needed in order to improve our knowledge about the important topic of the evaluation of quality of orthodontic treatment.

## 5. Conclusions

The quality of healthcare orthodontic treatment performed is extremely high: in fact, according to the general classification criteria of the index, to be considered as a high-standard treatment, the sample should have a minimum percentage of patients (up to 5%) which fall in the no improvement category, while a high percentage of them (at least 70%) should be part of the improved cases with a score reduction of at least 30% and more than 40% of patients should show a great improvement. Our results demonstrate meeting the criteria required for the high-standard therapy: 100% of the cases improved and 92% of them greatly improved.

In conclusion, the evaluation of orthodontic treatments performed by the postgraduate students enables to evaluate the skills acquired and, consequently, to reflect the quality of orthodontic university program.

## Figures and Tables

**Table 1:** Mean PAR distribution related with categorical variables: gender, technique, and extractions.

		Initial PAR Mean (CI 95%)	Final PAR Mean (CI 95%)	Change in PAR Score Mean (CI 95%)	Mann–Whitney U Test (*p* Value)	Change in PAR % Mean (CI 95%)	Mann–Whitney U Test (*p* Value)
Gender	Male (*n* = 28)	21.29 (18.06–24.52)	1.36 (0.27–2.45)	19.93 (16.71–23.15)	*p* = 0.4691	94.13 (89.77–98.49)	*p* = 0.6599
	Female (*n* = 22)	18.09 (14.92–21.26)	0.86 (0.03–1.69)	17.23 (14.38–20.08)		95.64 (92.04–99.24)	
Technique	Straight wire (*n* = 43)	19.44 (17.08–21.80)	1.05 (0.31–1.79)	18.40 (16.128–20.68)	*p* = 0.2481	95.18 (92.18–98.18)	*p* = 0.9656
	Self ligating (*n* = 7)	24.43 (16.84–32.02)	1.71 (−0.62–4.05)	22.71 (15.33–30.09)		92.43 (82.68–102.18)	
Extractions	Yes (*n* = 5)	26.60 (18.53–34.67)	2.40 (−0.74–5.54)	24.20 (17.93–30.47)	*p* = 0.0989	92.98 (84.06–101.89)	*p* = 0.5680

## Data Availability

All data are available upon request to corresponding Author.

## References

[1] Dyken R.A., Sadowsky P.L., Hurst D. (2001). Orthodontic outcomes assessment using the peer assessment rating index. Angle Orthod..

[2] Richmond S., Shaw W.C., O’Brien K.D., Buchanan I.B., Jones R., Stephens C.D., Roberts C.T., Andrews M. (1992). The development of the PAR Index (Peer Assessment Rating): Reliability and validity. Eur. J. Orthod..

[3] Onyeaso C.O., BeGole E.A. (2008). Associations between pretreatment age and treatment time with orthodontic treatment outcome: A comparison by means of two orthodontic indices. Hell Orthod. Rev..

[4] Hickman J.H. (1975). Directional edgewise orthodontic approach. J. Clin. Orthod..

[5] Firestone A.R., Häsler R.U., Ingervall B. (1999). Treatment results in dental school orthodontic patients in 1983 and 1993. Angle Orthod..

[6] Cook D.R., Harris E.F., Vaden J.L. (2005). Comparison of university and private- practice orthodontic treatment outcomes with the American Board of Orthodontics objective grading system. Am. J. Orthod. Dentofac. Orthop..

[7] Ramanathan C. (2006). PAR index in the evaluation of the stability of the orthodontic treatment results. A Review. Acta Med..

[8] Pangrazio-Kulbersh V., Kaczynski R., Shunock M. (1999). Early treatment outcome assessed by the Peer Assessment Rating index. Am. J. Orthod. Dentofac. Orthop..

[9] Templeton K.M., Powell R., Moore M.B., Williams A.C., Sandy R. (2006). Are the Peer Assessment Rating Index and the Index of Treatment Complexity, Outcome, and Need suitable measures for orthognathic outcomes?. Eur. J. Orthod..

[10] Gonzales-Gil-de-Bernabé P., Bellot-Arcis C., Montiel-Company J.M., Gandia-Franco J.L. (2014). Evaluation of treament outcomes in a 3 years post-graduate orthodontic program using the peer assessment rating (par). J. Clin. Exp. Dent..

[11] Richmond S., Shaw W.C., Roberts C.T., Andrews M. (1992). The PAR Index (Peer Assessment Rating): Methods to determine outcome of orthodontic treatment in terms of improvement and standards. Eur. J. Orthod..

[12] Bernas A.J., Banting D.W., Short L.L. (2007). Effectiveness of phase I orthodontic treatment in an undergraduate teaching clinic. J. Dent. Educ..

[13] Campbell C.L., Roberts W.E., Hartsfield J.K., Qi R. (2007). Treatment outcomes in a graduate orthodontic clinic for cases defined by the American Board of Orthodontics malocclusion categories. Am. J. Orthod. Dentofac. Orthop..

[14] Yang-Powers L.C., Sadowsky C., Rosenstein S., BeGole E.A. (2002). Treatment outcome in a graduate orthodontic clinic using the American Board of Orthodontics grading system. Am. J. Orthod. Dentofac. Orthop..

[15] Pinskaya Y.B., Hsieh T.J., Roberts W.E., Hartsfield J.K. (2004). Comprehensive clinical evaluation as an outcome assessment for a graduate orthodontics program. Am. J. Orthod. Dentofac. Orthop..

[16] Onyeaso C.O., Begole E.A. (2006). Orthodontic Treatment—Improvement and Standards using the Peer Assessment Rating Index. Angle Orthod..

[17] Egolf R.J., BeGole E.A., Upshaw H.S. (1990). Factors associated with orthodontic patient compliance with intraoral elastic and headgear wear. Am. J. Orthod. Dentofac. Orthop..

[18] Ngan P., Kess B., Wilson S. (1989). Perception of discomfort by patients undergoing orthodontic treatment. Am. J. Orthod. Dentofac. Orthop..

[19] Sergl H.G., Klages U., Zentner A. (1998). Pain and discomfort during orthodontic treatment: Causative factors and effects on compliance. Am. J. Orthod. Dentofac. Orthop..

[20] Blanck-Lubarsch M., Hohoff A., Wiechmann D., Stamm T. (2014). Orthodontic treatment of children/adolescents with special health care needs: An analysis of treatment lenght and clinical outcomes. BMC Oral Health.

[21] Vu C.Q., Roberts W.E., Hartsfield J.K., Ofner S. (2008). Treatment complexity index for assessing the relationship of treatment duration and outcomes in a graduate orthodontics clinic. Am. J. Orthod. Dentofac. Orthop..

[22] Brown P.N., Kulbersh R., Kaczynski R. (2011). Clinical outcomes assessment of consecutively finished patients in a 24-month orthodontic residency: A 5-year perspective. Am. J. Orthod. Dentofac. Orthop..

[23] Sfondrini M.F., Gandini P., Castroflorio T., Garino F., Mergati L., D’Anca K., Trovati F., Scribante A. (2018). Buccolingual Inclination Control of Upper Central Incisors of Aligners: A Comparison with Conventional and Self-Ligating Brackets. Biomed Res. Int..

[24] Holman J.K., Hans M.G., Nelson S., Powers M.P. (1998). An assessment of extraction versus nonextraction orthodontic treatment using the peer assessment rating (PAR) index. Angle Orthod..

[25] Beckwith F.R., Ackerman R.J., Cobb C.M., Tira D.E. (1999). An evaluation of factors affecting duration of orthodontic treatment. Am. J. Orthod. Dentofac. Orthop..

[26] Sfondrini M.F., Debiaggi M., Zara F., Brerra R., Comelli M., Bianchi M., Pollone S.R., Scribante A. (2012). Influence of lingual bracket position on microbial and periodontal parameters in vivo. J. Appl. Oral Sci..

[27] Tervonen M.M., Pirttiniemi P., Lahti S. (2011). Development of a measure for orthodontists to evaluate patient compliance. Am. J. Orthod. Dentofacial. Orthop..

[28] Karasiunok A.Y., Smahliuk L.V. (2018). The role of parents in motivation for orthodontic treatment for children. Wiad. Lek..

[29] Scribante A., Contreras-Bulnes R., Montasser M.A., Vallittu P.K. (2016). Orthodontics: Bracket Materials, Adhesives Systems, and Their Bond Strength. Biomed Res. Int..

[30] Willems G., Heidbüchel R., Verdonck A., Carels C. (2001). Treatment and standard evaluation using the Peer Assessment Rating Index. Clin. Oral Investig..

[31] Al Yami E.A., Kuijpers-Jagtman A.M., Van’t Hof M.A. (1998). Occlusal outcome of orthodontic treatment. Angle Orthod..

[32] Barenghi L., Barenghi A., Cadeo C., Di Blasio A. (2019). Innovation by Computer-Aided Design/Computer-Aided Manufacturing Technology: A Look at Infection Prevention in Dental Settings. Biomed Res. Int..

[33] Hosseinzadeh Nik T., Gholamrezaei E., Keshvad M.A. (2019). Facial asymmetry correction: From conventional orthognathic treatment to surgery-first approach. J. Dent. Res. Dent. Clin. Dent. Prospects..

[34] Scribante A., Sfondrini M.F., Gatti S., Gandini P. (2013). Disinclusion of unerupted teeth by mean of self-ligating brackets: Effect of blood contamination on shear bond strength. Med. Oral Patol. Oral Cir. Bucal.

[35] Lorente C., Lorente P., Perez-Vela M., Esquinas C., Lorente T. Orthodontic management of a complete and an incomplete maxillary canine-first premolar transposition. Angle Orthod..

[36] Sfondrini M.F., Gandini P., Malfatto M., Di Corato F., Trovati F., Scribante A. (2018). Computerized Casts for Orthodontic Purpose Using Powder-Free Intraoral Scanners: Accuracy, Execution Time, and Patient Feedback. Biomed Res. Int..

[37] Isola G., Matarese M., Briguglio F., Grassia V., Picciolo G., Fiorillo L., Matarese G. (2019). Effectiveness of Low-Level Laser Therapy during Tooth Movement: A Randomized Clinical Trial. Materials.

[38] Sfondrini M.F., Gandini P., Alcozer R., Vallittu P.K., Scribante A. (2018). Failure load and stress analysis of orthodontic miniscrews with different transmucosal collar diameter. J. Mech. Behav. Biomed. Mater..

[39] Sha H.N., Choi S.H., Yu H.S., Hwang C.J., Cha J.Y., Kim K.M. (2018). Debonding force and shear bond strength of an array of CAD/CAM-based customized orthodontic brackets, placed by indirect bonding- An In Vitro study. PLoS ONE.

